# Sarcoidosis Versus Lymphoma: A Clinical Diagnostic Dilemma in a Patient With Extensive Lymphadenopathy

**DOI:** 10.7759/cureus.43281

**Published:** 2023-08-10

**Authors:** Beisi Ji, Nazima Khatun, Elmira Mostafidi, Raavi Gupta, Samy I. McFarlane

**Affiliations:** 1 Internal Medicine, Downstate-Health Sciences University of New York, Downstate Medical Center, Brooklyn, USA; 2 Pathology, Downstate-Health Sciences University of New York, Downstate Medical Center, Brooklyn, USA; 3 Internal Medicine, Downstate-Health Sciences University of New York, Brooklyn, USA

**Keywords:** granulomatous disease, extensive lymphadenopathy, non-caseating granuloma, lymphoma, sarcoidosis

## Abstract

Sarcoidosis is a granulomatous disease involving multiple organ systems. In its classic form, sarcoidosis is associated with non-caseating granuloma. Several differential diagnostic entities exist for sarcoidosis, including tuberculosis (TB), as well as lymphomas. In this report, we present a case of sarcoidosis in a 55-year-old male with diffuse lymphadenopathy and hepatosplenic involvement, highlighting the differential diagnostic point for this disease.

## Introduction

Sarcoidosis is a multisystem disease with an unclear etiology with pulmonary and extrapulmonary manifestations. Extrapulmonary sarcoidosis can impact the lymph nodes, leading to lymphadenopathy, and systemic features may include fever, malaise, night sweats, and dry cough. Non-caseating granuloma is found to be most commonly associated with sarcoidosis [1]. Lymphomas are a heterogeneous group of malignancies originating from lymphocytes, often involving peripheral lymph nodes and presenting with B symptoms. The presence of overlapping B symptoms, such as weight loss, night sweats, and lymphadenopathy, in sarcoidosis and lymphoma might complicate the diagnosis process. Distinguishing between these two diseases may become even more challenging if the lymph node biopsy shows granuloma.

Here, we present a case of a 55-year-old male with initial clinical features and physical findings suggestive of lymphoma. Further investigation, including lymph node excisional biopsy, resulted in a diagnosis of sarcoidosis with extrapulmonary involvement.

## Case presentation

A 55-year-old male with diabetes was hospitalized for an infected diabetic foot ulcer. Initial vitals were unremarkable. Physical examination was significant for diffuse, palpable, non-tender lymphadenopathy and right second toe ulcer.

**Figure 1:**
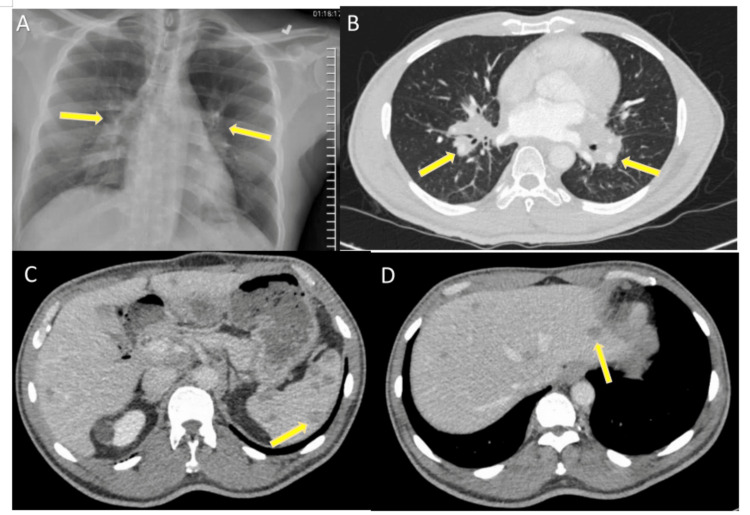
(A) Chest X-ray showing bilateral hilar fullness. (B) Chest CT with contrast showing confluent hilar/mediastinal soft tissue mass. (C,D) Abdominal CT with contrast showing innumerable hypodense lesions throughout the liver and spleen. Imaging findings are marked with yellow arrows. CT: computed tomography

Laboratory results showed a significant elevation of erythrocyte sedimentation rate (ESR) of 55 mm/hour (reference range: 0-20 mm/hour) and C-reactive protein (CRP) of 9 mg/L (reference range: 0-8 mg/L). Chest X-ray (Figure 1A) revealed bilateral hilar fullness. Computed tomography (CT) of the chest with contrast (Figure 1B) demonstrated left hilar calcified nodules with multisystem lymphadenopathy, including confluent hilar, mediastinum, tracheal, aortopulmonary, and bilateral axillary, suspicious for lymphoma.

On further history-taking, he reported unquantified unintentional weight loss and intermittent night sweats for the past three years. He denied fever, chills, cough, or other upper respiratory infection symptoms. He works as a bulldozer mechanic, lives with two dogs and nine cats, and denied tobacco, alcohol, and illicit drug use. He was born in the Caribbean and moved to the United States over 30 years ago, and traveled back and forth often.

CT of the abdomen and pelvis with contrast revealed innumerable hypodense lesions throughout the liver and spleen (Figure 1C, 1D) with multiple enlarged lymph nodes, including bilateral superficial inguinal region, left preaortic, para-aortic, and bilateral parasternal and precardiac region. CT of the neck with contrast showed enlarged, mildly enhancing submental nodes and smaller nodes in the submandibular areas bilaterally and supraclavicular regions.

**Figure 2:**
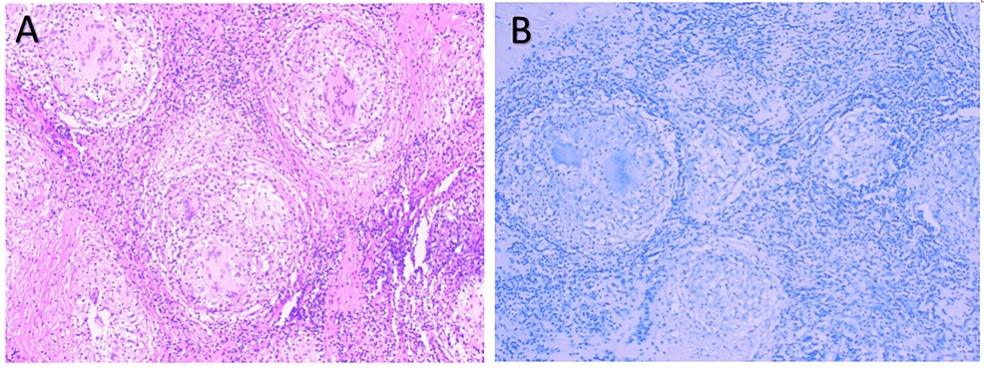
(A) Lymph node architecture was replaced by well-circumscribed non-necrotizing granulomas. These granulomas contain epithelioid histiocytes, some multinucleated giant cells, and a thin layer of surrounding lymphocytes (H&E: 40×). (B) AFB staining does not show any acid-fast bacilli within the granulomas (AFB: 40×). H&E: hematoxylin and eosin, AFB: acid-fast bacilli

Excisional biopsy of the submandibular lymph node revealed well-circumscribed epithelioid non-necrotizing granulomas with tightly packed epithelioid cells, some multinucleated giant cells, and lymphocytes. There was no histological evidence of lymphoma, and pathological findings were mostly consistent with sarcoidosis. Acid-fast staining revealed no bacilli within the granulomas. *Mycobacterium tuberculosis* (MTB) polymerase chain reaction (PCR) was negative (Figure 2). Further laboratory findings revealed an elevated serum angiotensin-converting enzyme level of 105.3 U/L (reference range: 9-67 U/L) with normal calcium of 9.7 mg/dL (reference range: 8.6-10.3 mg/dL) and 1,25-dihydroxyvitamin D levels of 29 pg/mL (reference range: 18-72 pg/mL). HIV, QuantiFeron tuberculosis (TB) gold, and histoplasma urine antigen results were negative (Table 1). The patient was discharged with a presumptive diagnosis of sarcoidosis with an outpatient follow-up at the pulmonology clinic.

**Table 1:** Routine laboratory findings during admission, including metabolic panel, blood count, HIV, and QuantiFeron TB gold. HIV: human immunodeficiency virus, TB: tuberculosis, CRP: C-reactive protein, ESR: erythrocyte sediment rate

Variable	During admission	Reference range
CRP (mg/L)	9	0-8
ESR (mm/hour)	55	0-20
Sodium (mmol/L)	137	136-145
Calcium (mg/dL)	9.7	8.2-10
Albumin (g/dL)	3.8	3.5-5.7
Alkaline phosphatase (U/L)	131	34-104
Aspartate aminotransferase (U/L)	15	13-39
Alanine aminotransferase (U/L)	16	7-52
Total bilirubin (mg/dL)	0.6	0.3-1
White cell count (K/uL)	3.78	3.5-10.8
Hemoglobin (g/dL)	13.1	14-18
Thyroid-stimulating hormone (uIU/mL)	0.55	0.38-4.7
25-hydroxy vitamin D (ng/mL)	8.1	30-95
1,25-dihydroxyvitamin D (pg/mL)	29	18-72
Angiotensin-converting enzyme (U/L)	105.3	9-67
QuantiFeron TB gold	Negative	
HIV antigen/antibody combination	Negative	
Histoplasma galactomannan antigen, urine (ng/mL)	<0.2	<0.2

## Discussion

Sarcoidosis is an inflammatory disease that involves multiple systems with pulmonary and extrapulmonary manifestations [1]. Pulmonary symptoms include dyspnea, nonproductive cough, and chest discomfort. Extrapulmonary sarcoidosis can cause generalized lymphadenopathy, skin involvement, arthralgias, and constitutional symptoms such as fatigue, fever, malaise, anorexia, night sweats, and unintentional weight loss [2]. Other systems involved in sarcoidosis are ocular, cardiovascular, and neurological systems, giving rise to nonspecific symptoms. The most prominent feature of sarcoidosis on chest radiography is bilateral hilar lymphadenopathy, with or without lung parenchyma involvement [3]. In sarcoidosis that has been present for two or three decades after onset, lymph node calcification can also be seen [4]. Furthermore, the abdominal viscera might be affected, with the liver being the most frequent. Multiple focal splenic lesions can also be found in 6%-33% of patients with sarcoidosis [5]. Sarcoidosis is often a diagnosis of exclusion in patients with suggestive clinical, radiographic, and tissue biopsy findings of non-caseating granuloma.

The clinical presentation of lymphoma could resemble sarcoidosis, and it might also affect the mediastinal lymph nodes, which leads to diagnostic confusion with sarcoidosis. Lymphoma is a tumor of the lymphoid system and is categorized into Hodgkin's lymphoma (HL) and non-HL (NHL) [6]. Typical symptoms include fatigue, diffuse painless lymphadenopathy, and B symptoms such as fever, night sweats, and weight loss [5]. It can also cause splenomegaly and metastasis to the liver, bone marrow, and lungs. Lymph node excisional biopsy is the gold standard diagnostic test. Reed-Sternberg (RS) or Hodgkin's cells are typically seen with HL. However, reports have shown that granulomatous reactions might mask lymphomas of various histological types [7].

Although sarcoidosis and lymphoma are two seemingly separate entities, there have been reports in the literature that sarcoidosis may occur together with lymphoma, mainly NHL, but usually, lymphoma occurs after sarcoidosis with a median interval of 24 months. It was named sarcoidosis-lymphoma syndrome by Brincker in 1986. It was also found that there is a fivefold increased risk of developing lymphoma among patients with chronic active sarcoidosis [8,9].

Tuberculosis (TB) is another common granulomatous disease. TB is a contagious disease caused by *Mycobacterium tuberculosis* (MTB). The major type of TB is pulmonary TB, while extrapulmonary TB accounts for approximately 20%-30% of all TB cases, with lymphadenitis being the most common form [10,11]. Constitutional symptoms such as fever, malaise, fatigue, night sweat, and weight loss are nonspecific and have similarities with sarcoidosis and lymphoma. Pulmonary TB tends to have a productive cough and hemoptysis, whereas sarcoidosis usually presents with a dry cough. The confirmation of suspected TB is made by sputum smear microscopy for acid-fast bacilli and sputum culture. Histopathologically, tuberculosis typically exhibits necrotizing granuloma [11]. Fluorescent staining or Ziehl-Neelsen (ZN) staining should be performed to identify acid-fast bacilli (AFB). Polymerase chain reaction (PCR) for MTB in tissue biopsy increases sensitivity and specificity [12,13].

Other differential diagnoses to consider include berylliosis, granulomatous arteritis, cat scratch disease, and fungal infection [14].

## Conclusions

Both lymphoma and sarcoidosis can present with B symptoms, diffuse lymphadenopathy, and hepatosplenic involvement. Differentiating these two diseases become more challenging, and rarely, lymphoma and sarcoidosis coexist. Although rare, sarcoidosis should be considered a differential diagnosis entity in the presence of lymphadenopathy and a clinical picture that is overall suggestive of lymphoma.

## References

[1] Petousi N, Mathew J, Thomas EC (2012). A patient presenting with generalised lympadenopathy--sarcoidosis, lymphoma or tuberculosis?. BMJ Case Rep.

[2] Bhattad PB, Jain V (2020). Diffuse sarcoidosis masquerading as widespread malignant disease: a rare case report and literature review. J Investig Med High Impact Case Rep.

[3] Lynch JP 3rd, Ma YL, Koss MN, White ES (2007). Pulmonary sarcoidosis. Semin Respir Crit Care Med.

[4] Nardi A, Brillet PY, Letoumelin P (2011). Stage IV sarcoidosis: comparison of survival with the general population and causes of death. Eur Respir J.

[5] Storck K, Brandstetter M, Keller U, Knopf A (2019). Clinical presentation and characteristics of lymphoma in the head and neck region. Head Face Med.

[6] Manjunatha BS, Gowramma R, Nagarajappa D, Tanveer A (2011). Extranodal non-Hodgkin's lymphoma presenting as gingival mass. J Indian Soc Periodontol.

[7] Wu CY, Wang RC, Chen BJ (2020). Granuloma with an underlying lymphoma: a diagnostic challenge and a wider histologic spectrum including adult T-cell leukemia/lymphoma. Appl Immunohistochem Mol Morphol.

[8] Brincker H (1986). The sarcoidosis-lymphoma syndrome. Br J Cancer.

[9] Maayan H, Ashkenazi Y, Nagler A, Izbicki G (2011). Sarcoidosis and lymphoma: case series and literature review. Sarcoidosis Vasc Diffuse Lung Dis.

[10] Gopalaswamy R, Dusthackeer VN, Kannayan S, Subbian S (2021). Extrapulmonary tuberculosis-an update on the diagnosis, treatment and drug resistance. J Respir.

[11] Eshete A, Zeyinudin A, Ali S, Abera S, Mohammed M (2011). M. tuberculosis in lymph node biopsy paraffin-embedded sections. Tuberc Res Treat.

[12] Goel MM, Ranjan V, Dhole TN, Srivastava AN, Mehrotra A, Kushwaha MR, Jain A (2001). Polymerase chain reaction vs. conventional diagnosis in fine needle aspirates of tuberculous lymph nodes. Acta Cytol.

[13] Rasool G, Siraj MR, Naseem N, Anjum S, Lateef W, Nagi AH (2017). Detection of acid fast bacilli in tuberculous lymph node tissue and touch preparations-a comparative study. J Tuberc Res.

[14] Mehta AC, Ali SR (2017). Mnemonic for the differential diagnosis of non-caseating granulomas. Sarcoidosis Vasc Diffuse Lung Dis.

